# Decoding the first mitogenomes of *Polycelis* (Platyhelminthes, Tricladida, Planariidae): genomic architecture, evolutionary dynamics, and phylogenomic implication

**DOI:** 10.1186/s12864-025-12467-z

**Published:** 2026-01-05

**Authors:** Ning Li, Yan-Kun Shen, De-Zeng Liu, Zi-Mei Dong, Guang-Wen Chen

**Affiliations:** College of Life Science, Henan Normal University, Xinxiang, Henan 453007 China

**Keywords:** *Polycelis*, Mitogenome, Comparative analysis, Phylogeny

## Abstract

**Background:**

The genus *Polycelis* is characterized by the arrangement of multiple eye spots along the anterior dorsal margin of the head. These freshwater planarians are predominantly distributed in high-altitude ecosystems of temperate and subarctic zones across the Northern Hemisphere. Despite their significance as ecological bioindicators and models for regeneration, *Polycelis* remains controversial in taxonomy and phylogeny due to a critical lack of molecular data. Mitochondrial genomes (mitogenomes) have emerged as powerful tools for resolving deep phylogenetic relationships and species boundaries in morphologically conserved taxa. In this study, we present the first comparative mitogenomic analyses of four *Polycelis* species and phylogenetic reconstructions within Tricladida.

**Results:**

Through next-generation sequencing, we successfully assembled four complete mitogenomes of *Polycelis* species. Each circular mitogenome contains 12 protein-coding genes (PCGs), 22 tRNA genes (tRNAs), 2 rRNA genes (rRNAs), and a non-coding region. Comparative genomic analyses revealed conservation in both gene arrangement and nucleotide compositions across the four species. The tandem repeat sequences and stem-loop structures were identified in their non-coding regions. Evolutionary analyses integrating nucleotide diversity (Pi), genetic distance, and Ka/Ks ratios across 12 PCGs demonstrated significant evolutionary heterogeneity: *cox1* showed relatively low evolutionary rate, while *nad6* displayed the highest sequence variability. Phylogenomic reconstruction using Bayesian inference (BI) and maximum likelihood (ML) methods based on 30 triclad mitogenomes, consistently resolved *Polycelis* as a monophyletic clade within Planariidae. Four *Polycelis* species exhibited synapomorphic gene rearrangements.

**Conclusions:**

This study firstly elucidates *Polycelis* mitogenome architecture, evolutionary dynamics, providing critical insights into the genomic basis of mitochondrial evolution, the utility of mitogenomic data for molecular taxonomy, and the phylogenetic position of *Polycelis* within Tricladida. Additionally, this research offers valuable references for conservation and utilization of *Polycelis* genetic resources.

**Supplementary Information:**

The online version contains supplementary material available at 10.1186/s12864-025-12467-z.

## Background

The genus *Polycelis* Ehrenberg, 1831 [1] (Platyhelminthes: Turbellaria: Tricladida: Planariidae) derives its specific epithet from the numerous eyespots arranged in two rows along the anterior margin of the dorsal cephalic region [2]. Morphological synapomorphies of this genus include, rounded or arched anterior cephalic margin; robust and elongated pharynx situated in the anterior portion of the posterior body half; genital pore positioned near the caudal extremity; and extremely reduced indistinct common atrium [3]. These flatworms typically inhabit cold, clean freshwater environments of springs, streams, and river source zones [4] and are the critical member of freshwater planarian. This taxon has emerged as an ideal model organism for toxicological and regenerative biology research due to its remarkable environmental contaminant sensitivity [5] and extraordinary regenerative capacity [6, 7]. However, taxonomic delineation and phylogenetic resolution within this clade remain constrained by methodological limitations and insufficient molecular datasets.

Taxonomy of *Polycelis* is mainly based on the external features (body colour and size, cephalic morphology, eye number), histological examinations (pharyngeal musculature types, male atrium muscular architecture, absence of adenodactyls, reproductive system characters), and chromosomal karyotypes. However, these approaches exhibit remarkable limitations. External features show the high degree of interspecific similarity and considerable intraspecific variation within *Polycelis* species. For instance, cephalic morphology may vary from arcuate to triangular configurations, with rounded auricular protrusions extending laterally. Moreover, the number of eyes exhibits significant plasticity, ranging from dozens to approximately two hundred across individuals or populations. The histological features can be unreliable in accurately distinguishing close species, having elevated taxonomic error rates, leading to the proliferation of synonyms [8]. Moreover, the taxonomic validity of several putative species remains uncertain. Additionally, histological analyses present a critical operational limitation: they require the examination of sexually mature specimens to characterize reproductive organ. However, specimens collected in the field usually lack sexually reproductive stages, resulting in the absence of critical reproductive structures essential for species identification, thereby precluding conventional taxonomic analyses based on histological criteria. In contrast, chromosomal karyotyping can be applied to asexual individuals, providing cellular genetic markers through the quantification of chromosome numbers and morphological characters, which offers a validated complementary approach for taxonomy within the genus *Polycelis* [9, 10]. However, chromosomal polymorphism has been documented in specific *Polycelis* species, including *P. nigra* Müller, 1774, *P. tenuis* Ijima, 1884, and *P. felina* Dalyell, 1814, which encompass polyploid and aneuploid cytotypes [10–12]. Therefore, the integration of novel datasets is expected to contribute to resolving taxonomic ambiguities within *Polycelis*.

Molecular taxonomic and phylogenetic research on *Polycelis* remains significantly limited. Among over 40 species, molecular data have been reported for only five [13–20]. Modern phylogenetic analyses of *Polycelis* are based primarily on single-gene markers (*cox1*, *18S* rRNA, or *28S* rRNA) to reconstruct the evolutionary relationships within Tricladida and to assess its taxonomic position among Platyhelminthes. However, phylogenetic incongruences persist at both familial and generic levels, particularly regarding the disputed placement of Planariidae within the order Tricladida and the unresolved sister-group relationships among *Polycelis*, *Phagocata*, and *Crenobia* within the family [21, 22].

The limited resolution of single-gene markers, as exemplified by *18S* rRNA [23] which is the predominant molecular marker used in *Polycelis* and Planariidae phylogenetics, underscores the need for multi-locus approaches. Consequently, phylogenetic analyses based on mitogenomes provide concatenated datasets of multiple genes and establish a robust framework to resolve intergeneric relationships within Planariidae (Tricladida) with improved topological accuracy.

The mitogenomes of Platyhelminthes are single-stranded circular DNA molecules, typically ranging in size from 14 kb to 21 kb. These mitogenomes generally consist of 12 protein-coding genes (PCGs), 22 transfer RNA genes (tRNAs), two ribosome RNA genes (rRNAs). The mitogenomic analyses can provide critical genome-scale insights into evolutionary features through base composition, gene arrangement, and genetic code deviations [24]. The mitogenomes are characterized by a simple genetic architecture, small size, maternal inheritance, high copy number, low recombination frequencies, and accelerated evolutionary rates [25]. Consequently, mitogenomic data have emerged as a powerful tool for species delimitation, phylogenetic reconstruction, molecular evolutionary studies, population structure analyses, and comparative genomics across diverse animal taxa [26, 27]. However, to date, only two mitogenomes—*Crenobia alpina* (Dana, 1766) and *Phagocata gracilis* (Haldeman, 1840)—have been reported within Planariidae, a family comprising 12 genera and approximately 190 species. Notably, the genus *Polycelis* comprising over 40 recognized species remains entirely unexplored in terms of complete mitogenomes annotation, representing a critical gap in comparative genomics and phylogenetic analysis for this taxonomically diverse group.

In this study, we firstly present the mitogenome sequences for four *Polycelis* species to address existing issues in the classification and phylogenetic relationships within the genus *Polycelis*. This study characterized the mitogenome structure, including gene arrangement, genome size, nucleotide composition, codon usage, tRNA secondary structures, gene overlaps, intergenic spacers, and non-coding regions. Comparative analysis by integrating these data with previously two published Planariidae mitogenomes facilitated the identification of effective molecular markers for molecular taxonomy and phylogenetic reconstruction within the genus *Polycelis* and among Planariidae genera [21, 22]. Additionally, phylogenetic analyses were conducted incorporating four *Polycelis* species and all 26 publicly available Tricladida mitogenomes (published or accessible via NCBI) to resolve inter-familial and inter-generic evolutionary relationships and clarify the phylogenetic position of *Polycelis* within the order [22, 28–42].

## Methods

### Sample collection and DNA extraction

The specimens of four *Polycelis* species, including two formally published species (*Polycelis nyingchica* Liu, 1994 and *P. yangchengensis* Dong, Chen, Zhang & Liu, 2017) and two undescribed species (*Polycelis* sp. NMA and *Polycelis* sp. QHY), were collected from freshwater habitats across China between 2021 and 2024. *Polycelis* sp. NMA was gathered in Nanma’an Village, Hui County, Xinxiang City, Henan Province (35.68°N, 113.61°E) on 5 October 2021. *Polycelis* sp. QHY was collected from a spring in Qinheyuan National Wetland Park, Qinyuan County, Changzhi City, Shanxi Province (36.79°N, 112.05°E) on 12 July 2022. The specimen of *P. nyingchica* [43] was obtained from a small stream in Zheba Village, Bayi District, Nyingchi City, Xizang Autonomous Region (29.82°N, 93.77°E) on 24 August 2023. *P. yangchengensis* [3] was found beneath a small waterfall in Hougou Village, Yangcheng County, Jincheng City, Shanxi Province (35.27°N, 112.15°E) on 5 May 2024. All collected specimens were maintained in a 10 °C incubator. Following sexual maturation, specimens underwent precise dissection of the pharyngeal and posterior genital regions for histological analysis to confirm species identification. Concurrently, anterior body fragments were cultured in the incubator to induce complete regeneration into morphologically intact individuals for subsequent mitochondrial DNA extraction [44]. The *Polycelis* specimens were submitted to Novogene Co., Ltd. (Beijing, China) for sequencing on the Illumina NovaSeq 6000 platform [45], which generated ≥ 5 GB of raw data.

### Morphological comparisons from histological analysis

The flatworms were relaxed by exposure to 1% nitric acid in a petri dish and then fixed in Bouin’s fluid for 24 h. After fixation, the specimens were rinsed in 70% ethanol, dehydrated in a graded ethanol series, cleared with xylene, and embedded in paraffin wax. The embedded blocks were sectioned serially at a thickness of 6 μm using a retracting rotary microtome. The sections were stained with either hematoxylin and eosin or Casson’s Mallory-Heidenhain stain [46]. Photographs were taken with a Leica digital camera attached to a compound microscope. Histological voucher specimens have been deposited at the Zoological Museum of Henan Normal University.

### Mitogenome assembly and annotation

The raw data were imported into VMware Workstation Pro v17.0.0 [47], and filtered using fastp [48], including trimming adapter sequences, removing high-N-rate sequences, short-lenth sequences, and low-quality reads, to obtain high-quality clean data. The Seqtk [49] was employed for extracting the mapped reads. The randomly selected mapped reads were used for mitogenome assembly via NOVOPlasty v4.3.3 [50], utilizing the *cox1* sequence of *Crenobia alpina* (Planariidae) as reference. The mitogenomes were annotated using MitoZ v3.6 [51] with genetic code 9 (Echinoderm Mitochondrial). Subsequently, further sequence analysis of the gene was aligned and annotated using Geneious Prime v8.1.3 [52]. The two rRNA genes (*rrnS* and *rrnL*) were manually annotated by aligning homologous regions with published mitogenomes of closely related species and with neighbouring genes. Unsuccessfully annotated protein-coding genes (PCGs) by MitoZ were identified through ORF prediction combined with homology database to manually validate the sequence boundaries. The 22 tRNAs and their secondary structures were predicted using the MITOS2 plugin [53] on the Galaxy Europe platform (http://usegalaxy.org) with genetic code 9. The comparative visualization of tRNA structures across four *Polycelis* species was performed using Adobe Illustrator 2023 [54].

### Genetic distance analysis of the *cox1* gene

To substantiate species delineation, we performed a genetic distance analysis of the mitochondrial *cox1* gene. The dataset integrated *cox1* sequences extracted from the four novel mitogenomes with additional *cox1* sequences amplified via PCR from 6 to 7 specimens per species to assess interspecific differences and intraspecific variation. The *cox1* gene was amplified using the universal primers BarTF (ATAGGTGGKTTTGGTAAT) and COIR (ATTWAYAWCAACACTACGAC) [55, 56] under standard PCR conditions. All sequences were aligned using MAFFT, and pairwise genetic distances were calculated under the Kimura 2-parameter (K2P) model with MEGA v11.0 [57].

### Comparative analysis and visualization of mitogenomes

The annotated mitogenomes was visualized as a circular map using OGDraw [58]. The mitogenomes of *Phagocata gracilis* and *Crenobia alpina* (Planariidae) were downloaded from NCBI (https://www.ncbi.nlm.nih.gov) for comparative genomic analyses. The relative synonymous codon usage (RSCU), nucleotide composition, and base composition skews (AT-skew = (A − T) / (A + T) and GC-skew = (G − C) / (G + C)) were analyzed using PhyloSuite v1.2.3 [59]. We conducted genomic evolutionary analyses using DnaSP v5.10.01 [60], including quantification of the nucleotide diversity (Pi) across PCGs, sliding window assessments (window size: 200 bp, step size: 20 bp) to detect variation distribution patterns, and evolutionary selection pressure evaluation through the ratio of non-synonymous (Ka) and synonymous substitutions rates (Ks) of PCGs. Genetic distances of the 12 PCGs were calculated using the Kimura 2-parameter (K2P) model in MEGA v11.0 [57]. Data visualization was performed using GraphPad Prism v9.0.0 [61] for A + T content, AT-skew, GC-skew, and genetic distances, while Ka/Ks ratios were plotted in OriginPro 2022 (OriginLab Corporation, Northampton, MA, USA). Tandem repeats of the non-coding region were identified using Tandem Repeats Finder [62], and their secondary structures were predicted by MFold software [63]. Comparative homology analysis of 6 complete mitogenomes was performed using BLAST + v2.16.0 [64], with alignment results visualized via the BLAST Ring Image Generator (BRIG) [65]. Further visualization of the particular sequence identity results was analyzed by the mVISTA Web Server [66].

### Gene rearrangement analysis

The ancestral gene order of Tricladida was reconstructed using the MLGO web server [67]. The inferred ancestral gene arrangement was visualized with the gene order of known Tricladida species and analyzed for mitochondrial gene rearrangements. The reference tree used to map gene arrangements was modified based on phylogenetic analyses.

### Phylogenetic analyses

Four newly sequenced *Polycelis* mitogenomes and 26 publicly available mitogenomes from four families within Tricladida (Planariidae, Dugesiidae, Dendrocoelidae, Kenkiidae), retrieved from NCBI were chosen as the ingroup (Table 1), and two Polycladida flatworm species, *Prosthiostomum siphunculus* and *Planocera reticulata* were selected as outgroups [68, 69].

**Table 1:** Details of the species and mitogenomes used in this study

Order	Family	Species	Size(bp)	Accession no.	Resource
Tricladida	Geoplanidae	*Bipalium adventitium*	15,494	MZ561467	[36]
		*Bipalium admarginatum*	18,990	NC072986	[40]
		*Bipalium kewense*	15,666	NC045216	[30]
		*Bipalium vagum*	17,149	MZ561468	[36]
		*Vermiviatum covidum*	15,540	MZ561472	[36]
		*Diversibipalium mayottensis*	15,989	MZ561470	[36]
		*Diversibipalium multilineatum*	15,660	MZ561469	[36]
		*Arthurdendyus triangulatus*	20,309	NC085462	[32]
		*Parakontikia atrata*	16,513	NC068631	[34]
		*Parakontikia ventrolineata*	17,210	MT081960	[29]
		*Amaga expatria*	14,962	NC057980	[38]
		*Amaga* sp.	14,909	PP727122	[37]
		*Obama* sp. MAP-2014	14,909	KP208777	[22]
		*Rhynchodemus sylvaticus*	16,891	PQ468469	[42]
		*Platydemus manokwari*	19,959	MT081580	[31]
		*Microplana scharffi*	15,291	PP711816	[33]
	Dugesiidae	*Schmidtea mediterranea*	16,562	KM115583	Unpublished
		*Girardia tigrina*	15,938	MW972220	[28]
		*Girardia* sp. ER-2015	15,951	KP090061	[21]
		*Dugesia constrictiva*	17,634	OK078614	[44]
		*Dugesia ryukyuensis*	17,015	AB618488	[39]
		*Dugesia japonica*	17,799	AB618487	[39]
	Planariidae	*Crenobia alpina*	16,894	KP208776	[22]
		*Phagocata gracilis*	18,759	KP090060	[21]
		*Polycelis yangchengensis*	14,714	PV946914	This study
		*Polycelis nyingchica*	16,029	PV946912	This study
		*Polycelis* sp. QHY	16,839	PV946913	This study
		*Polycelis* sp. NMA	16,623	PV946911	This study
	Uteriporidae	*Miroplana shenzhensis*	14,344	NC062124	[35]
		*Obrimoposthia wandeli*	15,185	NC050050	[41]
Polycladia	Prosthiostomidae	*Prosthiostomum siphunculus*	15,181	KT363736	[65]
	Planoceridae	*Planocera reticulata*	15,486	LC503531	[66]

Thirty-six genes (12 PCGs, 2 rRNAs, and 22 tRNAs) were extracted from the mitogenomes using PhyloSuite (v1.2.3). The extracted 12 PCGs, two rRNAs, and 22 tRNAs were aligned separately using MAFFT [70], employing distinct algorithms based on gene type: the L-INS-i algorithm for PCGs and the E-INS-i algorithm for rRNAs and tRNAs. After alignment, ambiguous sites were removed using Gblocks [71]. The genes used for phylogenetic tree construction were concatenated using the “Concatenate Sequence” module built into PhyloSuite [59], and the output was saved in “Phylip” format for subsequent tree construction.

Phylogenetic trees were reconstructed for Tricladida using Bayesian inference (BI) and maximum likelihood (ML) analyses. Five datasets were generated for phylogenetic analyses: (1) P12R: 12 PCGs excluding third codon position + 2 rRNAs (12,311 bp), (2) P123: 12 PCGs (14,133 bp), (3) P123R: 12 PCGs + 2 rRNAs (17,022 bp), (4) P123RT: 12 PCGs + 2 rRNAs + 22 tRNAs (19,301 bp), (5) P123-AA: amino acid sequences of the 12 PCGs. The best-fit substitution models and partitioning schemes [see Additional file 1] for the five datasets were inferred using PartitionFinder 2 [72]. Bayesian analysis was performed using MrBayes v3.2.6 [73] with running 2 × 10^6^ generations of MCMC and sampling every 1,000 generations. Convergence of independent runs was assessed using a standard deviation of split frequencies < 0.01 in MrBayes v3.2.6 and effective sample size (ESS) values > 200 in Tracer v1.7 [74]. The first 25% of samples were discarded as burn-in, and the remaining samples were used to generate the tree and estimate the posterior probabilities (PP). The best-fit substitution model [see Additional file 2] was selected using ModelFinder [75], which was subsequently implemented in IQ-TREE [76] for Maximum likelihood (ML) tree construction with 5,000 ultrafast bootstrap replicates to assess the reliability of the internal branches [35, 46]. The phylogenetic trees were annotated and visualized using ITOL [77] and FigTree v1.4.4 [78].

## Results

### Morphological differences for species diagnosis

Our comparative morphological analysis of the reproductive apparatus [see Additional file 3] identified clear and consistent diagnostic characters among the four *Polycelis* species. *P. nyingchica* is characterized by an elongated male atrium with the penis papilla positioned distant from the gonopore and the penis bulb occupying only half of the dorso-ventral space. In contrast, the other three species exhibit shorter male atrium with the penis papilla extending to or protruding through the gonopore and substantially larger penis bulbs filling over three-quarters of the dorso-ventral space. A key diagnostic feature of *P. yangchengensis* is the vague boundary between its penis papilla and penis bulb, combined with the absence of a distinct seminal. In contrast, the other three species are characterized by clearly asymmetrical dorsal and ventral penis papilla lips and well-developed seminal vesicles that occupy one-third or more of the penis bulb space. Furthermore, *Polycelis* sp. QHY can be readily distinguished by its extremely slender bursal canal. This characteristic serves as a critical diagnostic feature distinguishing it from its closely related congener, *Polycelis* sp. NMA.

The combination of these stable morphological differences across multiple reproductive structures provides robust morphological evidence for recognizing them as distinct species.

### Genetic distance analysis of the *cox1* gene

Genetic distance analysis performed on a 786 bp alignment of the mitochondrial *cox1* gene revealed significant molecular divergence among the four putative *Polycelis* species [see Additional file 4]. The intraspecific genetic variation was remarkably low in all four species: *P. yangchengensis* (0–0.26%), *Polycelis* sp. NMA (0–0.26%), *Polycelis* sp. QHY (0–0.38%), and *P. nyingchica* (0–1.0%). In contrast, interspecific distances revealed substantial differentiation, with values ranging from 3.8% to 14.0%. Notably, the minimum interspecific distance (3.8–4.5%) was observed between *Polycelis* sp. QHY and *Polycelis* sp. NMA, while the maximum divergence (13.5–14.0%) occurred between *P. nyingchica* and *Polycelis* sp. NMA. The minimum interspecific distance (3.8–4.5%) was substantially greater than the maximum intraspecific distance (1.0%).

The consistent and significant discontinuities in genetic distances, with all interspecific values markedly higher than intraspecific variation, provide robust molecular evidence for recognizing them as distinct species. These molecular data are fully congruent with the morphological evidence, jointly validating the taxonomic hypothesis of four separate species within the *Polycelis*.

### Basic structure and nucleotide composition

**Fig. 1:**
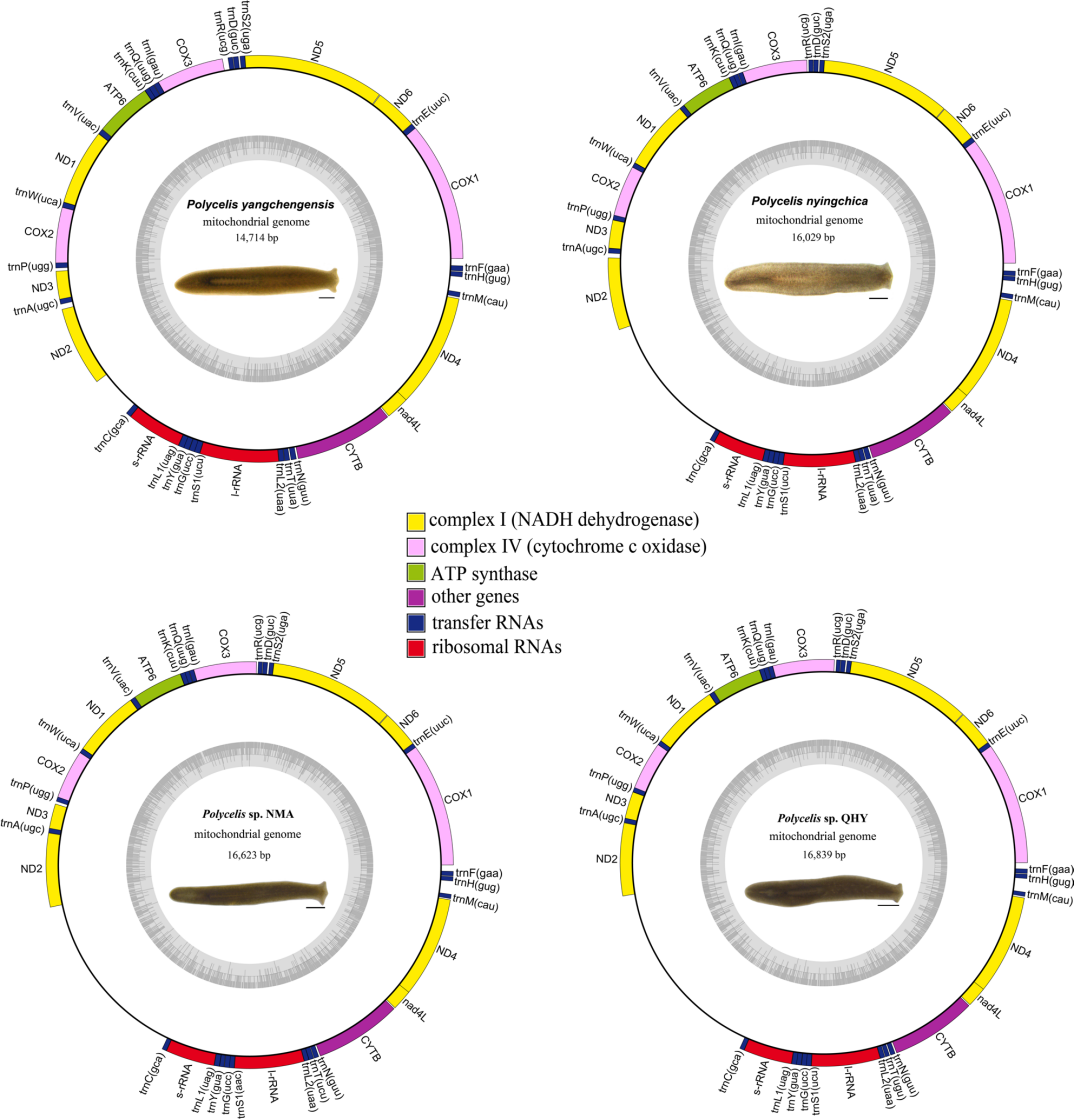
Mitochondrial genome arrangements of four *Polycelis* species. Scale bar: 500 μm

The newly sequenced complete mitogenomes of *Polycelis* are typical circular, single-stranded structures. The mitogenomes of all four species contained 12 protein-coding genes (PCGs), 22 tRNAs, 2 rRNAs, and one non-coding region, with identical gene arrangement patterns (Fig. 1). Mitogenomes vary in lengths from 14,714 bp in *P. yangchengensis* to 16,839 bp in *Polycelis* sp. QHY, with *P. nyingchica* (16,029 bp) and *Polycelis* sp. NMA (16,623 bp) exhibiting intermediate sizes. These size variations were primarily attributed to differences in the non-coding region lengths.

**Fig. 2:**
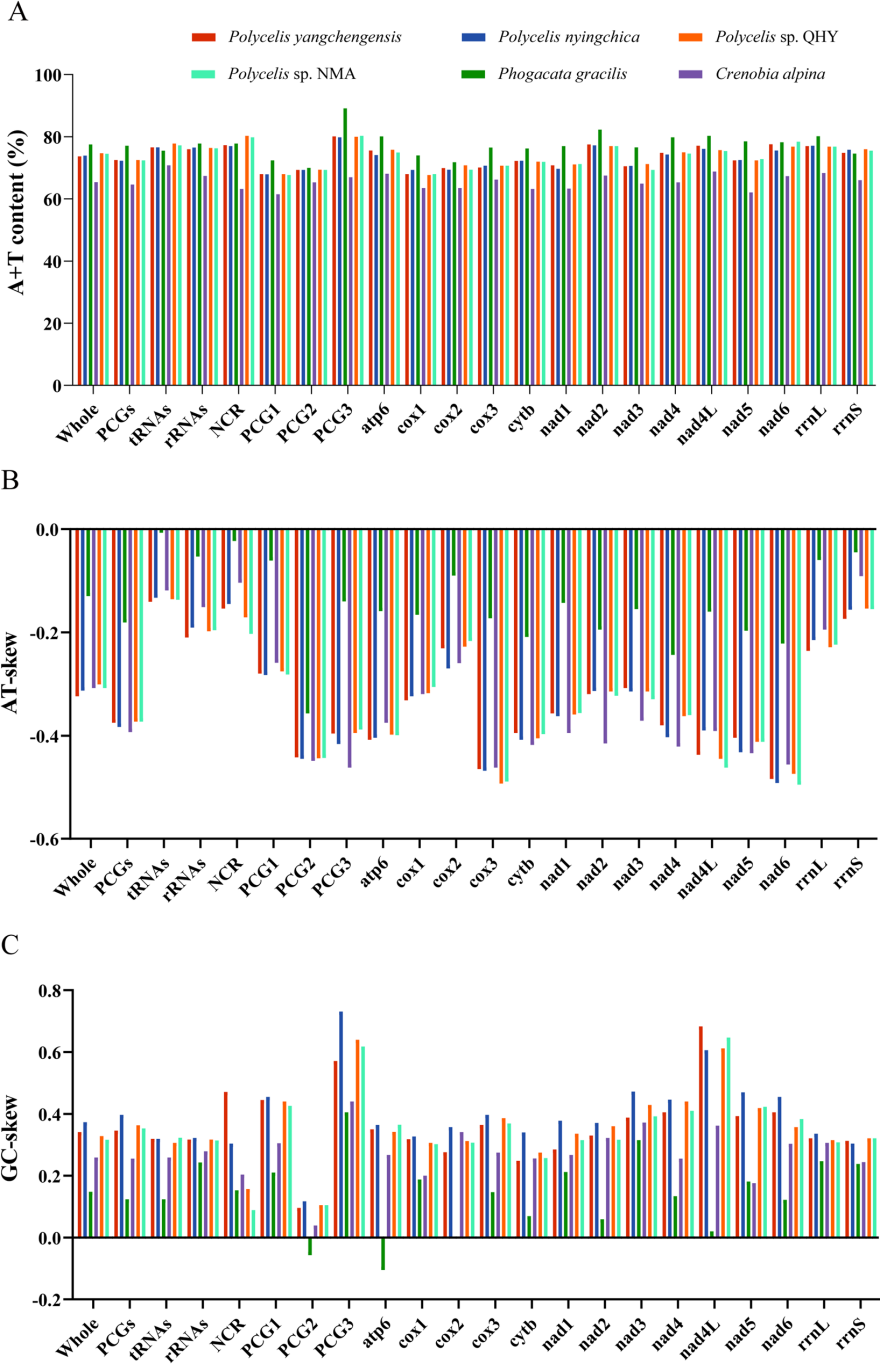
Comparison ofthe A + T contents, nucleotide skewness of six Planariidae species. **A **A + T content; **B **AT-skew; **C **GC-skew. PCG1, the first codon position; PCG2, the second codon position; PCG3, the third codon position; Whole, the complete sequences of whole mitogenome; NCR, Non-coding region

The mitogenomes of *Polycelis* showed strong AT bias, ranging from 73.7% (*P. yangchengensis*) to 74.7% (*Polycelis* sp. QHY), with *P. nyingchica* and *Polycelis* sp. NMA showing intermediate values of 73.9% and 74.5%, respectively [see Additional file 5]. The high A + T content in all four species was determined by their PCGs, rRNAs, tRNAs, and non-coding regions. Among these regions, the non-coding regions exhibited the highest A + T content (77.0%–80.3%), specifically 77.0% in *P. nyingchica*, 77.3% in *P. yangchengensis*, 79.8% in *Polycelis* sp. NMA, and 80.3% in *Polycelis* sp. QHY. Comparative analysis with other Planariidae species, *Crenobia alpina* consistently displayed lower A + T content than other species, while *Phagocata gracilis* showed higher A + T content in its PCGs, rRNAs, and entire genome. However, *C. alpina* and *Ph. gracilis* exhibited lower tRNA A + T content compared to the four *Polycelis* species (Fig. 2A). Analysis of the 12 PCGs revealed that *nad2* had the highest A + T content, while *cox1* showed the lowest across the *Polycelis*.

Nucleotide skew analysis revealed consistently negative AT-skew and positive GC-skew values across all four *Polycelis* mitogenomes (Fig. 2B, C), indicating similar nucleotide bias patterns among these species. Nucleotide skew analysis across the six Planariidae species revealed that *Ph. gracilis* had the slightly negative AT-skew, while *C. alpina* showed the pronouncedly negative AT-skew closer to *Polycelis* species, with lower similarity than that observed among *Polycelis* congeners (Fig. 2B). *Ph. gracilis* exhibited significantly lower GC-skew than other Planariidae species. Except for weakly negative values in PCG2 and *atp6*, and neutral skew in *cox2*, all other regions showed slightly positive GC-skew. The GC-skew values of *C. alpina* were exclusively within the range observed in four *Polycelis* species for its non-coding regions and protein-coding genes *cox2*, *nad2*, and *cytb*, while all other regions exhibited lower GC-skew values than those of the four *Polycelis* species (Fig. 2C).

### Protein-coding genes and condon usage

The PCGs measured 10,827 bp in *P. yangchengensis* and 10,899 bp in *P. nyingchica*, while those in *Polycelis* sp. QHY and *Polycelis* sp. NMA were 10,938 bp and 10,911 bp, respectively [see Additional file 5].

The newly sequenced *Polycelis* mitogenomes contained both standard ATN (ATG/ATT) and non-standard (GTG/TTG) initiation codons. These non-standard initiation codons occurred in seven PCGs (*cox2*, *cox3*, *atp6*, *nad2*, *nad3*, *nad4L*, *nad6*). TTG functioned as the initiation codon for *nad6*, *nad4L*, and *cox2* in all four *Polycelis* species, and for *cox3* in three species (excluding *Polycelis* sp. NMA). GTG served as the initiation codon for *nad3* in all species except *Polycelis* sp. QHY, and for *nad2* in *P. yangchengensis* and *P. nyingchica*. The most prevalent initiation codon was ATG, which occurred in five PCGs across all specimens. The termination codons consisted of complete TAA/TAG and incomplete “T-” or “TA-” [see Additional file 6]. TAA occurred more frequently than TAG, with “T-” typically serving as the termination codon for *cox1* and “TA-” for *nad3*.

**Fig. 3:**
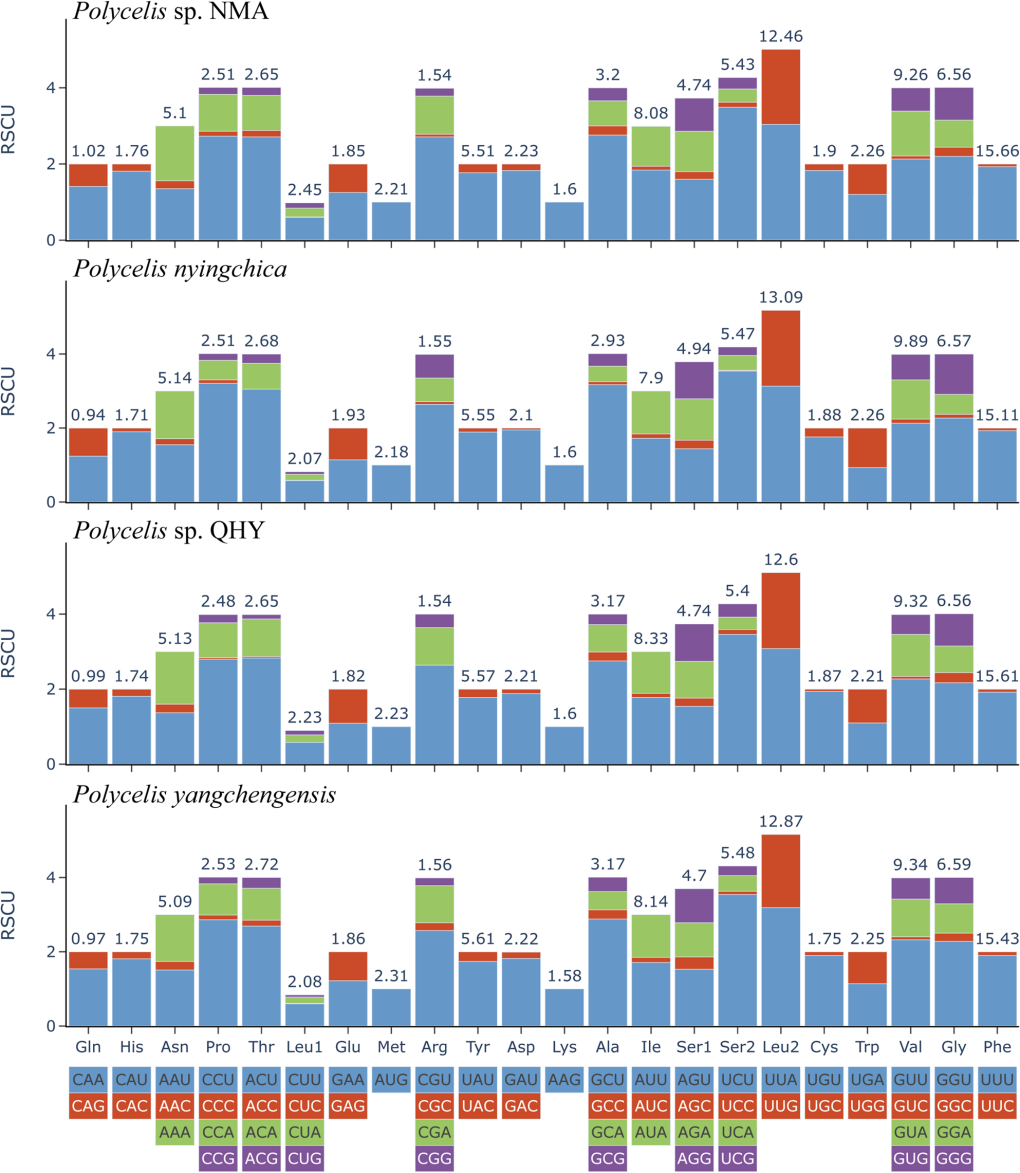
Relative synonymous codon usage (RSCU) of the mitogenomes of four *Polycelis* species

The four *Polycelis* species exhibited similar amino acid composition and relative synonymous codon usage (RSCU) patterns in their mitogenomes (Fig. 3). The total number of codons in PCGs ranged from 3,598 to 3,627 [see Additional file 7]. The three most frequently used codons were UUA (Leu2), AUU (Ile), and UUU (Phe), all consisting exclusively of A/U bases (Fig. 3). A/T nucleotides occurred more frequently than G/C at the third codon position, reflecting the strong A + T bias in the mitochondrial PCGs among *Polycelis*.

### Transfer and ribosomal RNA genes

**Fig. 4:**
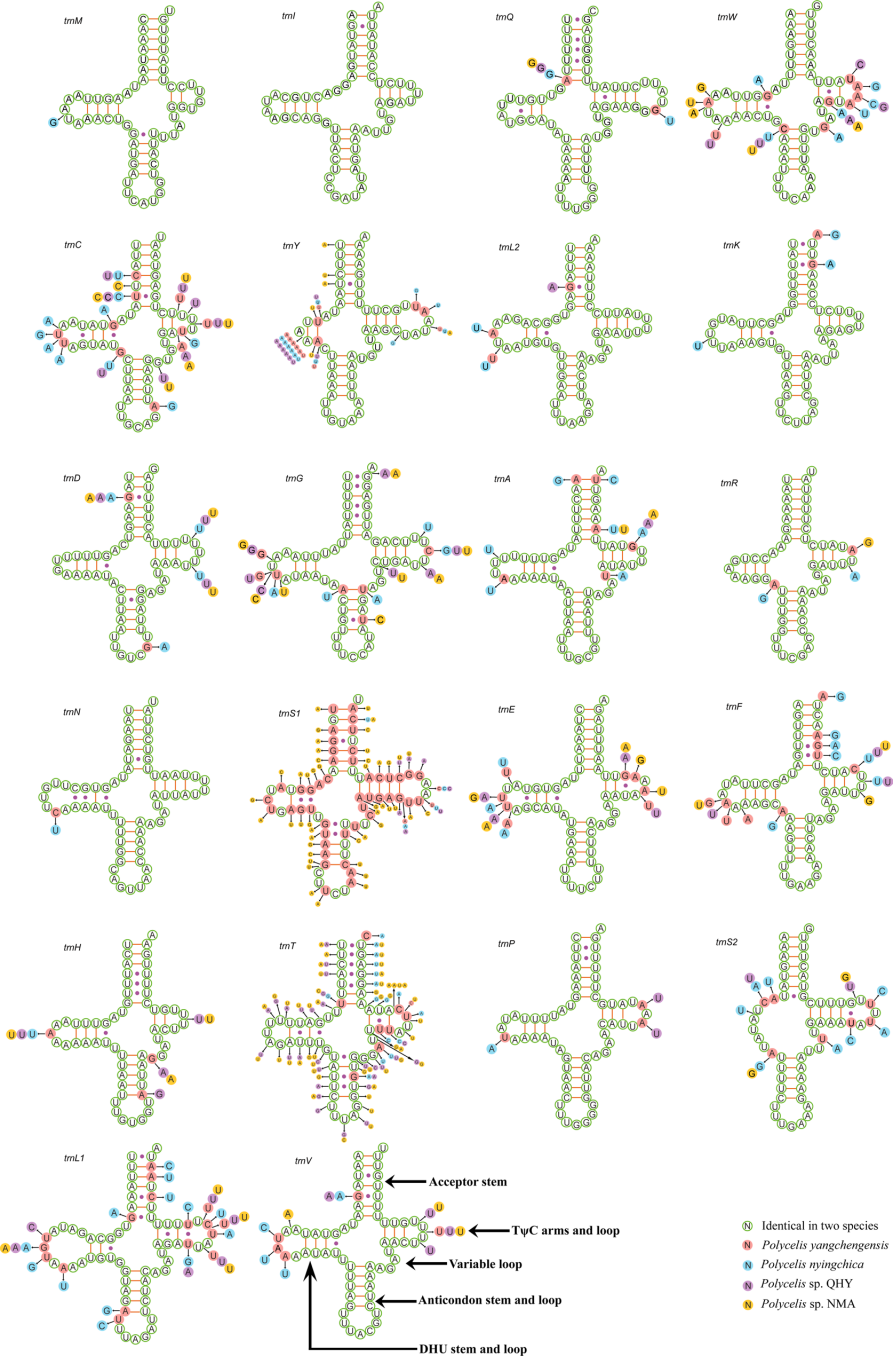
Secondary structure for the tRNAs of four *Polycelis* species

Twenty-two transfer RNA genes (tRNAs) of four sequenced *Polycelis* mitogenomes dispersed throughout the entire mitogenome (Fig. 1). The tRNA regions were 1,411 bp in both *Polycelis* sp. QHY and *Polycelis* sp. NMA, while *P. yangchengensis* and *P. nyingchica* showed slightly shorter lengths of 1,409 bp and 1,406 bp, respectively [see Additional file 5]. The 22 tRNAs varied in size from 58 bp (*trnR*) to 72 bp (*trnL1*) [see Additional file 6]. Based on the secondary structure, 21 of 22 tRNAs could fold into canonical cloverleaf secondary structures, while *trnS2* lacked the DHU arm, forming only a simple loop (Fig. 4). Comparative analysis of tRNAs across the four species revealed higher nucleotide substitution rates in *trnC*, *trnT*, *trnL1*, and *trnS1* relative to other tRNAs. The TΨC and DHU arms exhibited greater variability compared to the anticodon loop. Based on predicted secondary structures, six types of mismatched base pairs (A-A, A-C, A-G, G-U, U-C, and U-U) were identified across the four mitogenomes.

All four mitogenomes contained only four tRNA genes located between the two rRNA genes (*rrnL* and *rrnS*). The *rrnL* gene was 919–921 bp, while *rrnS* ranged from 647 to 651 bp across the four mitogenomes. The *rrnS* gene was located between *trnC* and *trnL1*, while *rrnL* was positioned between *trnS1* and *trnL2* [see Additional file 6].

### Overlapping sequences and intergenic spacers

Gene overlaps were identified at 13 loci across the four mitogenomes. All four species exhibited seven identical gene overlap regions [see Additional file 6]: *nad5-trnS2* (2 bp), *cox3-trnI* (2 bp), *trnQ-trnK* (11 bp), *trnK-atp6* (1 bp), *trnW-cox2* (3 bp), *cox2-trnP* (5 bp), and *nad4L-nad4* (32 bp). Intergenic spacers were identified at 12 loci, ranging from 1 to 171 bp in length. The length of intergenic spacers exhibited greater variability compared to overlapping regions.

### Non-coding region

In the mitogenomes of four *Polycelis* flatworms, the non-coding region located between *nad2* and *trnC* exhibited marked length variation. *Polycelis* sp. QHY possessed the longest region (2,628 bp), while *P. yangchengensis* displayed the shortest (493 bp). The corresponding lengths in *P. nyingchica* and *Polycelis* sp. NMA were 1,821 bp and 2,425 bp [see Additional file 5].

**Fig. 5:**
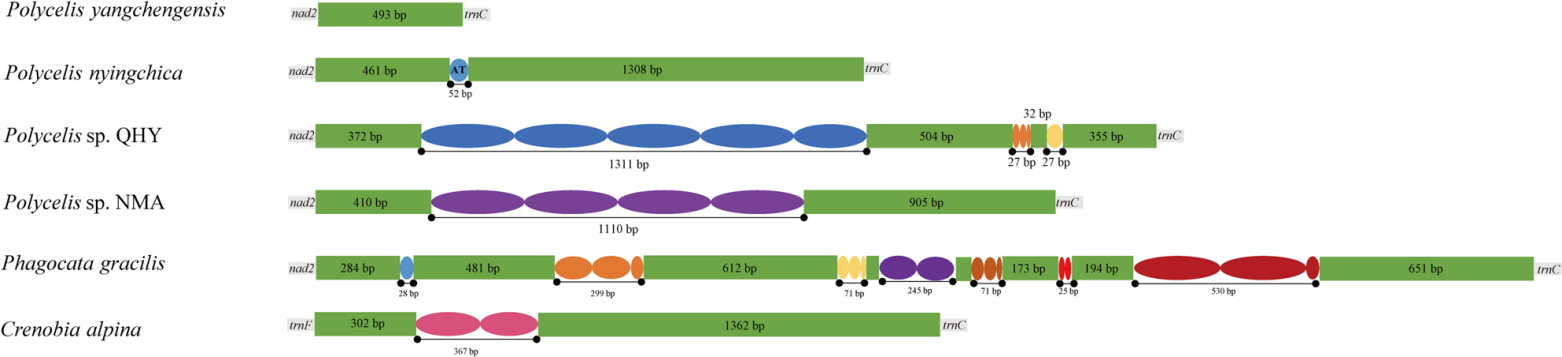
Organization of the control region in Planariidae mitochondrial genomes. The colored ovals indicate the tandem repeats; the remaining regions are shown with green boxes

This study identified various tandem repeat elements in the non-coding region of *Polycelis* mitogenomes. All species except *P. yangchengensis* exhibited specific tandem repeats. Both *Polycelis* sp. QHY and *Polycelis* sp. NMA possessed extended tandem repeats, measuring 1,311 bp and 1,110 bp, respectively. The other tandem repeat units were smaller, ranging from 27 to 32 bp. Variations in repeat unit length and copy number across *Polycelis* species resulted in different sizes of their non-coding regions. In addition to the four sequenced *Polycelis* mitogenomes, two additional Planariidae species (*Ph. gracilis* and *C. alpina*) were also found to have specific tandem repeats ranging in size from 25 bp to 530 bp (Fig. 5). Potential stem-loop secondary structures were identified in the non-coding regions of all six species [see Additional file 8].

### Nucleotide diversity and evolutionary rate

**Fig. 6:**
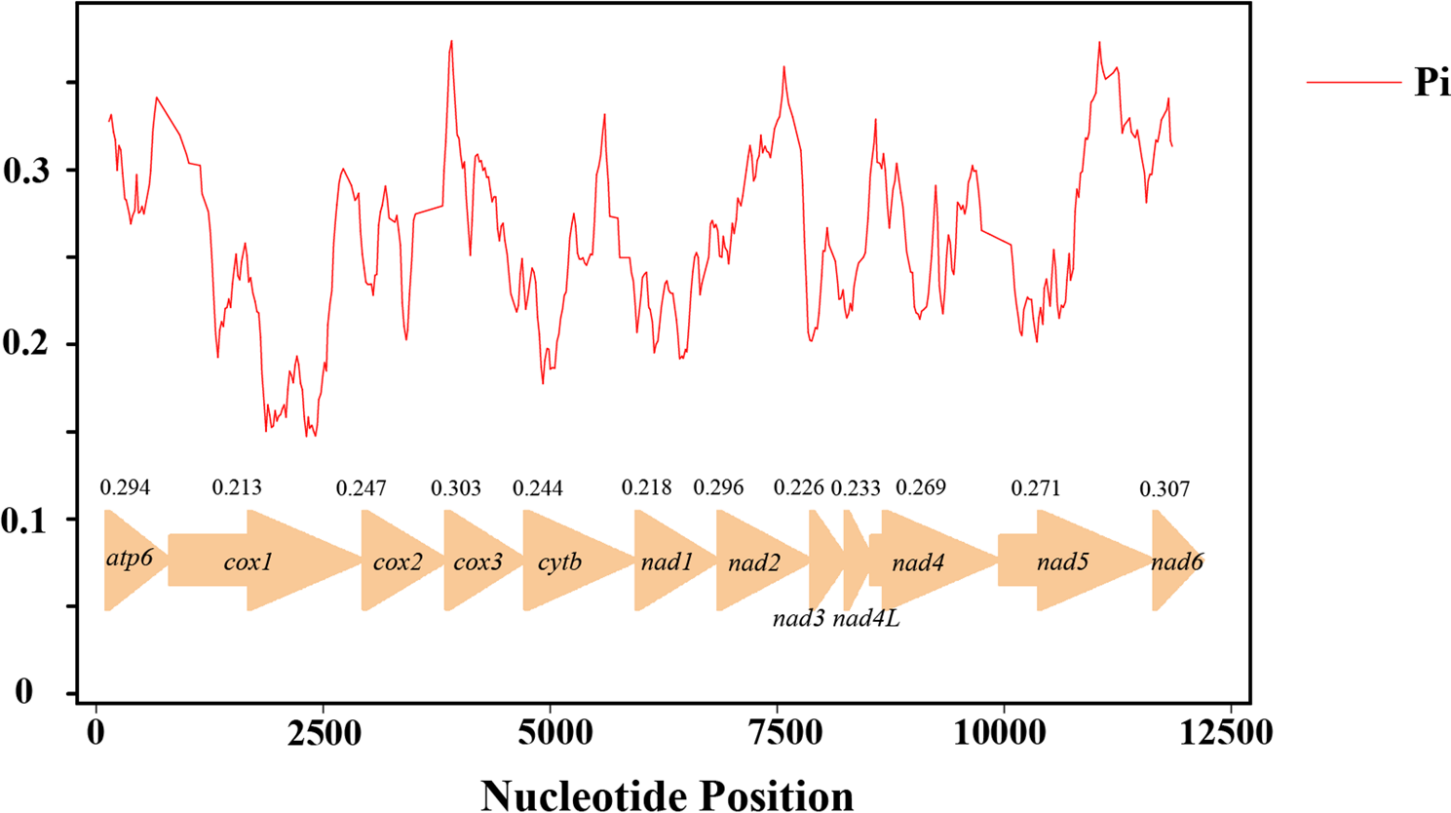
sliding window analysis of protein-coding genes of the six Planariidae species. The red curve shows the value of nucleotide diversity (Pi). Pi value of each PCGs was shown above the arrows

For comprehensive analysis, the four sequenced *Polycelis* mitogenomes were compared with two additional Planariidae species (*Ph. gracilis* and *C. alpina*). Sliding window analysis of 12 PCGs across six Planariidae mitogenomes revealed variable nucleotide diversity: *nad6* (0.307), *cox3* (0.303), *nad2* (0.296), and *atp6* (0.294) showed higher values, while *cox1* (0.213), *nad1* (0.218), *nad3* (0.226), and *nad4L* (0.233) exhibited lower diversity (Fig. 6). Nucleotide diversity (Pi) analysis of the four *Polycelis* species showed slightly different patterns compared to the six Planariidae species analysis, with relatively higher values in *nad6* (0.111), *nad2* (0.104), *nad5* (0.101), and *cox3* (0.0978), and lower diversity in *nad4L* (0.0604), *cox2* (0.0742), *nad3* (0.0777), and *cox1* (0.0821) [see Additional file 9].

**Fig. 7:**
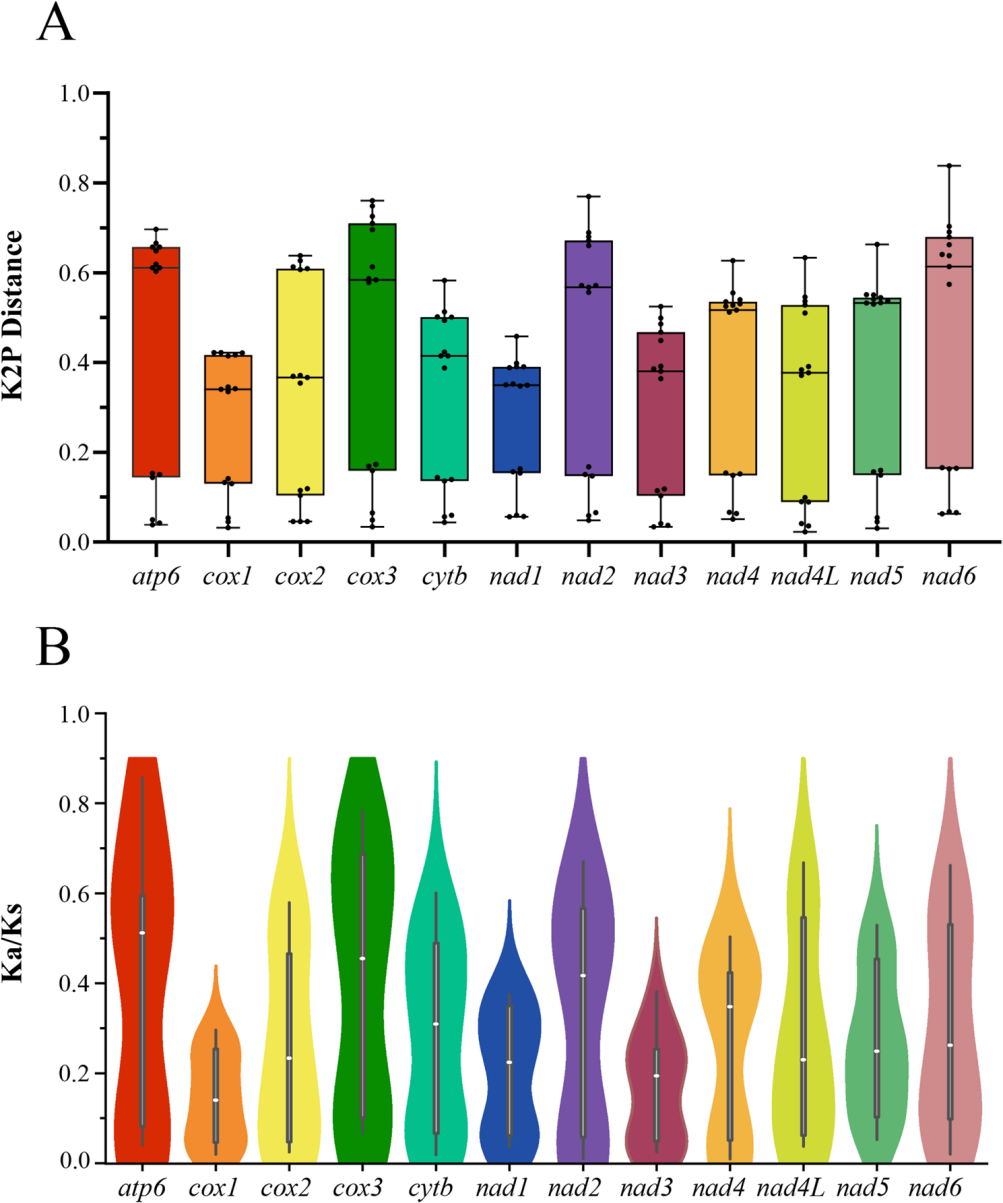
**A **Genetic distance of each protein-coding gene among six Planariidae species; **B **Ratio ofnon-synonymous (Ka) to synonymous (Ks) substitution rates of each protein-coding gene among six Planariidae species

Pairwise genetic distance analysis revealed similar inconsistencies between intra-generic and inter-generic comparisons within Planariidae. Among the six species from three genera in Planariidae, *nad6* (0.449), *cox3* (0.443), *nad2* (0.425), and *atp6* (0.423) exhibited the highest average genetic distances (Fig. 7A), indicating faster evolutionary rates in these four genes. Conversely, *cox1* showed the lowest average K2P distance (0.266), followed by *nad1* (0.293), *nad3* (0.271), and *nad4L* (0.310) [see Additional file 10], indicating these genes are relatively conserved. The average interspecific K2P distances among the four *Polycelis* species revealed higher values for *nad6* (0.122), *nad2* (0.116), *nad5* (0.108), and *cox3* (0.107), while *nad4L* showed the lowest distance (0.0633), followed by *cox2* (0.0789), *nad3* (0.0864), and *cox1* (0.0878) [see Additional file 11].

The pairwise Ka/Ks analyses showed that the average Ka/Ks ratios (ω) of 12 PCGs are from 0.141 to 0.406 (0 < ω < 1), indicating pervasive purifying selection across these protein-coding genes (Fig. 7B). The genes *cox3* (0.406), *atp6* (0.400), *nad2* (0.329), and *nad6* (0.297) exhibited relatively higher Ka/Ks ratios, while *cox1* (0.141), *nad3* (0.164), *nad1* (0.203), and *cox2* (0.258) showed lower values [see Additional file 12]. Ka/Ks analysis across *Polycelis* species revealed that while the highest (*nad5*: 0.108) and lowest (*nad4*: 0.0358) ω values differed from those observed in the six Planariidae species comparison [see Additional file 13]. All 12 PCGs of *Polycelis* consistently exhibited ω values within the range of 0 < ω < 1.

### Sequence identity analysis

**Fig. 8:**
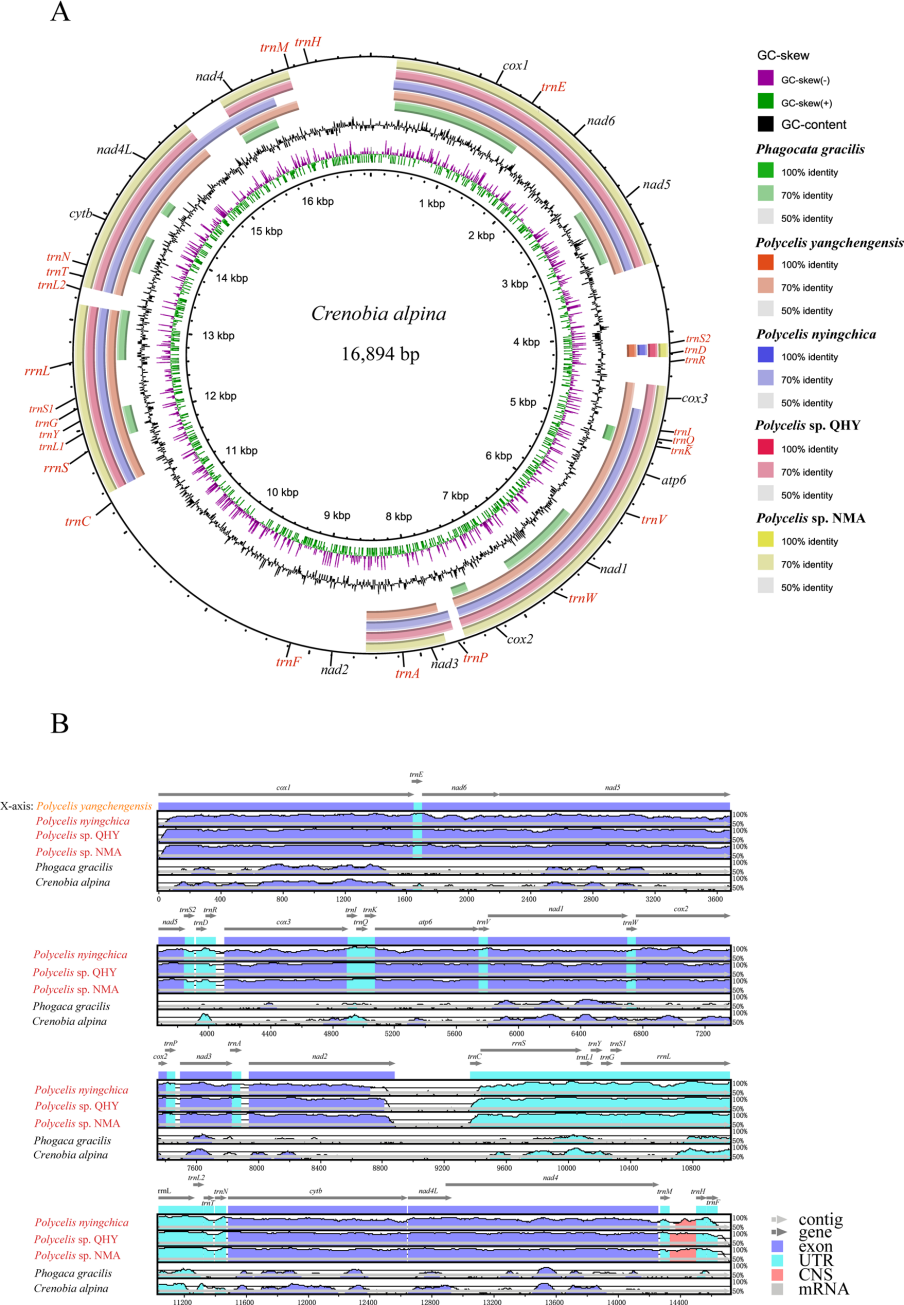
**A** BLAST comparison of six Planariidae mitogenomes. The comparison has been made with the *Crenobia alpina* as the reference species. In the outermost ring, the protein-coding genes are labelled in black, and other genesare labelled in red; **B **Major intragroup sequence identity variation visualized by BLAST alignment. The alignment was done against *Polycelis yangchengensis*. The names of *Polycelis* in the same group are in red

Multiple sequence alignment of the six Planariidae mitogenomes revealed significant sequence identity within the *Polycelis* genus. Using *C. alpina* as the reference genome, BLAST analysis revealed the following the order from innermost to outermost: *Ph. gracilis*, *P. nyingchica*, *P. yangchengensis*, *Polycelis* sp. QHY, and *Polycelis* sp. NMA (Fig. 8A). Specifically, relatively high and stable sequence identity was found in the protein-coding regions, with pairwise comparisons among *Polycelis* species consistently exceeding 75% identity. The mVISTA alignment (Fig. 8B) demonstrated high gene sequence homology among the four *Polycelis* species, with significantly higher intra-generic than inter-generic sequence identity.

### Gene rearrangement

**Fig. 9:**
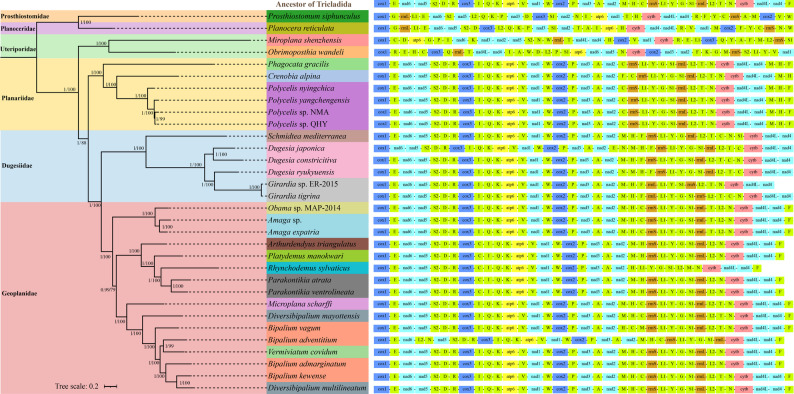
Phylogenetic tree produced by Bayesian inference based on the dataset of P123RT. Numerals at nodes are Bayesian posterior probabilities (PP) and bootstrap support values (BS), respectively

We incorporated the four newly sequenced species and 26 publicly available Tricladida sequences from NCBI to compare gene arrangements across 19 genera from 4 families with ancestral Tricladida. Among the 30 analyzed species, we identified 12 distinct gene rearrangement patterns, including translocations, local inversions, and gene deletions (Fig. 9).

Within the family Uteriporidae, the genera *Miroplana* and *Obrimoposthia* exhibited two distinct gene rearrangement patterns, both showing extensive divergence from the ancestral Tricladida arrangement. All PCGs and tRNAs underwent substantial reorganization.

The four *Polycelis* species exhibited identical gene orders, and the genetic pattern is relatively conservative. Within the family Planariidae, the genera *Polycelis* and *Phagocata* share identical gene order, whereas *Crenobia* exhibits a distinct rearrangement involving translocation of *trnF*: the original *nad2-trnF-trnC* cluster is rearranged to *trnH-trnF-cox1*. Comparative analysis revealed that *Polycelis*, *Crenobia*, and *Phagocata* maintained ancestral Tricladida PCG arrangements, with only rearrangements of tRNA genes present. In *Polycelis* and *Phagocata*, *trnM-trnH* is displaced between *nad4* and *trnF*, whereas in *Crenobia*, *trnF* and *trnM-trnH* exchanged positions.

The Dugesiidae family exhibited five distinct gene rearrangement patterns across *Dugesia*, *Schmidtea*, and *Girardia* genera. The *Schmidtea* mitogenome exhibited tRNA rearrangements relative to ancestral Tricladida, with *trnF* shifting from *nad4-cox1* to *trnH-rrnS*, *trnS1* relocating from *trnG-rrnL* to *trnN-cytb*, and *trnC* moving from *trnH-rrnS* to *trnT-trnN*. *Girardia* sp. ER-2015 lacked both *trnG* and *trnC* genes compared to *G. tigrina*, while maintaining identical PCG arrangements. Compared to the ancestral Tricladida gene arrangement, *trnF* relocated from between *nad4* and *cox1* to between *trnH* and *rrnL*, while *trnC* shifted from between *trnH* and *rrnS* to between *trnT* and *trnN*. The three *Dugesia* species exhibited three distinct gene arrangements, primarily differing in *trnE* and *trnN* positions, while maintaining identical PCG organization relative to ancestral Tricladida, except for some tRNAs that have changed position.

Within Geoplanidae, ten genera exhibited four distinct gene rearrangement patterns. *Microplana*, *Diversibipalium*, and two *Bipalium* species (*B. vagum* and *B. kewense*) maintained the ancestral gene order. The gene order of *Obama* is consistent with that of *Amaga*, and they exhibit a transposition of *trnT* and *trnL2* relative to the ancestral arrangement. The genera *Arthurdendyus*, *Platydemus*, and *Parakontikia* shared identical gene arrangements, exhibiting two modifications from the ancestral pattern: loss of *trnT* and translocation of *trnC* from between *trnH* and *rrnS* to between *cox3* and *trnI*. *Vermiviatum* and *Bipalium admarginatum* have the same gene order, exhibiting complete conservation of the ancestral arrangement except for the loss of *trnT*. *Bipalium adventitium* differed from both the ancestral Tricladida arrangement and three other *Bipalium* species by the translocation of *trnL2-trnN* from between *rrnL* and *cytb* to between *trnE* and *nad5*, along with the absence of *trnT*.

### Phylogenetic analysis

Three distinct phylogenetic trees were generated using BI and ML analyses of five datasets (P12R, P123, P123R, P123RT, and P123-AA). The tree topologies derived from the four datasets P12R, P123, P123R, and P123RT under both BI and ML methods were highly consistent and showed strong nodal support (Fig. 9). In contrast, the trees inferred from the P123-AA dataset exhibited certain topological discrepancies in the relationships among the four families [see Additional file 14].

The ML and BI phylogenetic analyses based on the four datasets (P12R, P123, P123R, P123RT) consistently support Uteriporidae (Maricola) as the earliest diverging lineage within Tricladida (BS = 88, PP = 1) (Fig. 9). Planariidae diverged next, followed by the sister groups of Dugesiidae and Geoplanidae (BS = 100, PP = 1). The phylogenetic relationships among the four Tricladida families were supported as: (Uteriporidae + (Planariidae + (Dugesiidae + Geoplanidae))). The ML phylogenetic analysis based on P123-AA dataset indicated that Planariidae was the earliest diverging lineage within Tricladida [see Additional file 14 A]. Uteriporidae diverged next, followed by the sister groups of Dugesiidae and Geoplanidae (BS = 100). The phylogenetic relationships among the four families were inferred as: (Planariidae + (Uteriporidae + (Dugesiidae + Geoplanidae))). The BI phylogenetic analysis based on P123-AA dataset suggested a sister relationship between Uteriporidae and Planariidae, as well as between Dugesiidae and Geoplanidae, although both relationships with low support (PP = 0.5) [see Additional file 14B]. The overall phylogenetic relationships among the four families of Tricladida were reconstructed as follows: ((Planariidae + Uteriporidae) + (Dugesiidae + Geoplanidae)).

*Phagocata* was the basal lineage among the three genera of Planariidae (Fig. 9). The four *Polycelis* species formed a monophyletic clade sister to *Crenobia* with strong support (BS = 100, PP = 1). Within *Polycelis*, *P. nyingchica* from the Qinghai-Tibet Plateau represents the earliest-diverging basal lineage (BS = 100, PP = 1). The remaining species form a highly supported clade (BS = 100, PP = 1) comprising three endemic the Taihang Mountain taxa, within which *Polycelis* sp. NMA and *Polycelis* sp. QHY are sister species.

In Dugesiidae, *Schmidtea* formed a sister clade to *Dugesia* + *Girardia* with (BS = 100, PP = 1). The phylogeny revealed that *Girardia tigrina* and *Girardia* sp. ER-2015 were conspecific, forming a sister group to *Dugesia ryukyuensis* (BS = 100, PP = 1). *Dugesia japonica* and *D. constrictiva* were sister groups (BS = 100, PP = 1). This topology confirmed the paraphyletic status of *Dugesia* relative to *Girardia* (Fig. 9).

The three phylogenetic trees generated in this study show that Dugesiidae and Geoplanidae form sister groups. The monophyletic clade (*Obama* + *Amaga*) formed a sister group to all other Geoplanidae genera (BS = 100, PP = 1) (Fig. 9). For the P123RT dataset, BI and ML analyses support that the clade including *Arthurdendyus*, *Platydemus*, *Rhynchodemus*, and *Parakontikia* and the clade including *Microplana*, *Diversibipalium*, and *Bipalium* are each recovered as monophyletic, and these two monophyletic clades are sister to each other with moderate support (BS = 79, PP = 0.99). Notably, this topological arrangement demonstrates the paraphyly of *Bipalium*, as members of *Bipalium* are not clustered into a single exclusive clade.

## Discussion

### Features of mitogenome structure

The *Polycelis* mitogenomes exhibited high conservation in gene content, order, length, and nucleotide composition. Their genomic architecture followed the same organizational pattern observed in other Planariidae species. Nucleotide content and skewness analyses demonstrated strong AT bias, uniformly negative AT-skew and positive GC-skew across all four *Polycelis* species. This pattern reflects a genomic asymmetry with A + T content significantly exceeding G + C content, more A than T and more C than G, as previously reported in other planarians [21, 79].

Annotation of four sequenced mitogenomes revealed a universal absence of *atp8* gene. Annotation challenges for *atp8* stemmed from its rapid substitution rate and extreme divergence [80]. Previous studies have frequently reported *atp8* gene loss in flatworm populations [22, 68, 81, 82]. However, recent studies have detected potential *atp8* signals in flatworms through manual annotation and transcriptomic analyses [83]. Despite replicating the published *atp8*-detection methods, we discerned no transmembrane domain signals nor any corroborating evidence of *atp8* in *Polycelis* mitogenomes. Notably, the putative *atp8* sequences reported previously were all located in non-coding region [84], inconsistent with canonical *atp8* genomic positions in most metazoans. Thus, our study could not definitively identify the *atp8* gene, a limitation that may be resolved by future advances in sequencing and analytical methodologies.

In all four *Polycelis* specimens, *trnS2* formed a simple loop structure due to the absence of a dihydrouridine (DHU) arm, consistent with observations in other Planariidae species [21, 22], and common in planarian mitogenomes [85]. All four *Polycelis* mitogenomes contained mismatched base pairs in tRNA stem regions, a widespread phenomenon among planarians [21, 22]. These mismatches may originate from anomalous base pairing [86, 87], undergo RNA-editing correction [88], or potentially enhance tRNA functional efficiency as suggested by recent studies [79, 89].

Two non-canonical initiation codons (GTG and TTG) were identified in all four *Polycelis* mitogenomes. Additionally, non-canonical codons TAT and TTA have been reported in Platyhelminthes [24]. These four atypical initiation codons were predominantly found in *cox1*, *nad1*, *nad2*, and *nad5*. The incomplete termination codons “TA-” and “T-” are commonly utilized in *cox1*, *cox3*, *nad3*, and *nad5* genes of *Polycelis*. These truncated codons may affect gene expression levels or potentially be converted to complete TAA stop codons through post-transcriptional polyadenylation during mRNA maturation [90, 91].

### Mitogenome sequence characteristics and nucleotide diversity

The *Polycelis* mitogenomes contained a major non-coding region ranging in size from 1,493 bp to 2,628 bp with relatively high nucleotide substitution rates. This region showed significant length and compositional variation across species, consistent with its characterization as the most variable mitogenomic region [92–94]. Tandem repeats in non-coding region, documented for the first time in Planariidae mitogenomes, are frequently found in planarian and other metazoan mitogenomes [95]. Variation in tandem repeat copy number and length among species and individuals directly contributes to non-coding length polymorphism, resulting in mitogenome size variation. Stem-loop structures were identified in planarian non-coding regions, this feature also reported in other platyhelminths, suggesting potential roles in replication and transcription initiation [96, 97].

Nucleotide diversity analysis facilitates the assessment of genetic differentiation among populations and aids in designing species-specific markers [98, 99], particularly for species identification and phylogenetic studies in *Polycelis*. The *cox1* gene widely adopted as a universal barcode for animal species identification [100], exhibits relatively low variability within the *Polycelis* genus. But it represents the most evolutionarily conserved and least variable PCGs among the six Planariidae species. If the resolution power of *cox1* was proved to be low, genes with sufficient large size, fast evolution, and elevated Ka/Ks ratios should be considered as potential marker candidates [98, 101].

### Gene arrangement

Gene rearrangements were frequently observed in Tricladida mitogenomes, primarily involving tRNA positional changes. Gene order analysis revealed identical arrangements between *Polycelis* and *Phagocata*, but translocation events in *Crenobia*, suggesting its independent evolutionary trajectory within Planariidae. Distinct gene orders have proven highly diagnostic for delineating animal taxa across multiple taxonomic levels [102, 103]. The gene rearrangement pattern in *Crenobia* suggests that the genus should be an independent lineage within Planariidae. This specific feature of *Crenobia* likely resulted from independent mitogenomic evolutionary events. The gene arrangement in the genera *Microplana*, *Diversibipalium*, and two *Bipalium* species (*B. vagum*, *B. kewense*) aligns with the ancestral gene order, suggesting a conservative evolutionary divergence. The three *Dugesia* species exhibit gene rearrangements compared to the ancestral sequence, with distinct gene orders among species, suggesting relatively independent evolution. However, more representative mitogenomes from diverse populations are needed to validate this hypothesis.

### Phylogenetic relationship

The ML and BI phylogenetic analyses based on P123-AA dataset reconstructed the relationship of four families, contradicting previous studies [44, 104]. Our finding that marine planarians form a clade with terrestrial and freshwater planarians. The phylogenetic position of four families within Tricladida in our study aligns with previous findings [44, 104] based on four datasets (P12R, P123, P123R, and P123RT). The results reaffirm freshwater planarians as a paraphyletic group, revealing that the freshwater species of Dugesiidae show closer phylogenetic affinity to terrestrial planarians than to other freshwater planarians.

In this study, we analyzed two published *Girardia* species, which formed a strongly supported clade (BS = 100, PP = 1) but did not cluster as sister groups with *Dugesia*. Although previous studies generally support *Girardia* and *Dugesia* as sister groups, and a monophyletic *Dugesia* [24, 105], our findings indicate that *Dugesia* is paraphyletic [29]. Such divergence arises from differing specimen datasets. Blastn analysis of the *cox1* gene from our complete mitogenomes of *Girardia tigrina* showed highest similarity to *Dugesia* species in NCBI. Notably, it exhibited approximately 30% genetic distance from published *G. tigrina cox1* sequences, far exceeding normal intraspecific variation. We suspect that taxonomic misidentification due to the lack of copulatory apparatus evidence may explain these phylogenetic inconsistencies, underscoring the need for further validation. The mitogenome releases for certain planarian species usually did not specify whether species identification was based on valid reproductive organ evidence or merely on superficial morphological traits and colour patterns [21, 29–34]. It is therefore essential to include morphological evidence when sequencing planarians for complete mitogenomes.

In the family Planariidae, phylogenetic analysis incorporating newly sequenced *Polycelis* mitogenomes revealed *Phagocata* as earliest diverging genus, whereas *Polycelis* and *Crenobia* formed a sister group. This contrasts with previous *18S* rDNA-based phylogenies, which supported *Phagocata* and *Polycelis* as sister taxa [15]. These discrepancies indicate persistent limitations in elucidating the phylogenetic relationships within Tricladida, necessitating mitogenome sequencing of additional validated species and further population genetic and phylogenetic analyses [106].

Both mitochondrial phylogenomic analysis and BLAST comparisons revealed that the four *Polycelis* species formed a strongly supported clade with high sequence identity. The three *Polycelis* species from the Taihang Mountains exhibit noticeably shorter branches in phylogenetic trees compared to other congeners. Despite their shallow phylogenetic divergence, these species are clearly distinguishable by morphological differences in the reproductive apparatus, corroborated by interspecific genetic distances in the *cox1* gene reaching 3.8–6.7%, well within the range expected for distinct species. This pattern likely results from a recent evolutionary event in the Taihang Mountains.

## Conclusions

In this study, we sequenced and assembled four *Polycelis* mitogenomes, representing the first comprehensive comparative analysis of mitogenome for Planariidae. The comparative analysis revealed that *Polycelis* has strong AT bias that is identical to other Planariidae. There were differences in condon usage preferences among four *Polycelis* species. The Ka/Ks values of *Polycelis* were quantitatively lower than those reported for Planariidae, suggesting stronger purifying selection in the genes of the *Polycelis*. Based on the combined results of sliding window analysis, genetic distance calculation, and Ka/Ks ratio assessment, *cox3* and *nad6* may serve as potential DNA markers for species delineation in *Polycelis* and other Planariidae. Comparison of ancestral gene arrangements with those of 30 sequenced Tricladida species revealed 12 different patterns of gene rearrangement. Furthermore, the phylogenetic analysis results provide critical insights into the phylogenetic position of the *Polycelis* within Tricladida. This study provides a theoretical basis for inferring taxonomic characteristics of Planarian species and informing biodiversity conservation strategies in China.

## Supplementary Information

Supplementary Material 1.

Supplementary Material 2.

Supplementary Material 3.

Supplementary Material 4.

Supplementary Material 5.

Supplementary Material 6.

Supplementary Material 7.

Supplementary Material 8.

Supplementary Material 9.

Supplementary Material 10.

Supplementary Material 11.

Supplementary Material 12.

Supplementary Material 13.

Supplementary Material 14.

## Data Availability

The genome sequence data that support the findings of this study are openly available in GenBank of NCBI at ( https://www.ncbi.nlm.nih.gov/ ) under accession numbers: PV946911, PV946912, PV946913 and PV946914.
