# Supplementary figures and images for: Decoding the first mitogenomes of Polycelis (Platyhelminthes, Tricladida, Planariidae): genomic architecture, evolutionary dynamics, and phylogenomic implication
Source: BMC Genomics. 2026 Jan 5;27:130. doi: 10.1186/s12864-025-12467-z (PMC12870015; doi:10.1186/s12864-025-12467-z)

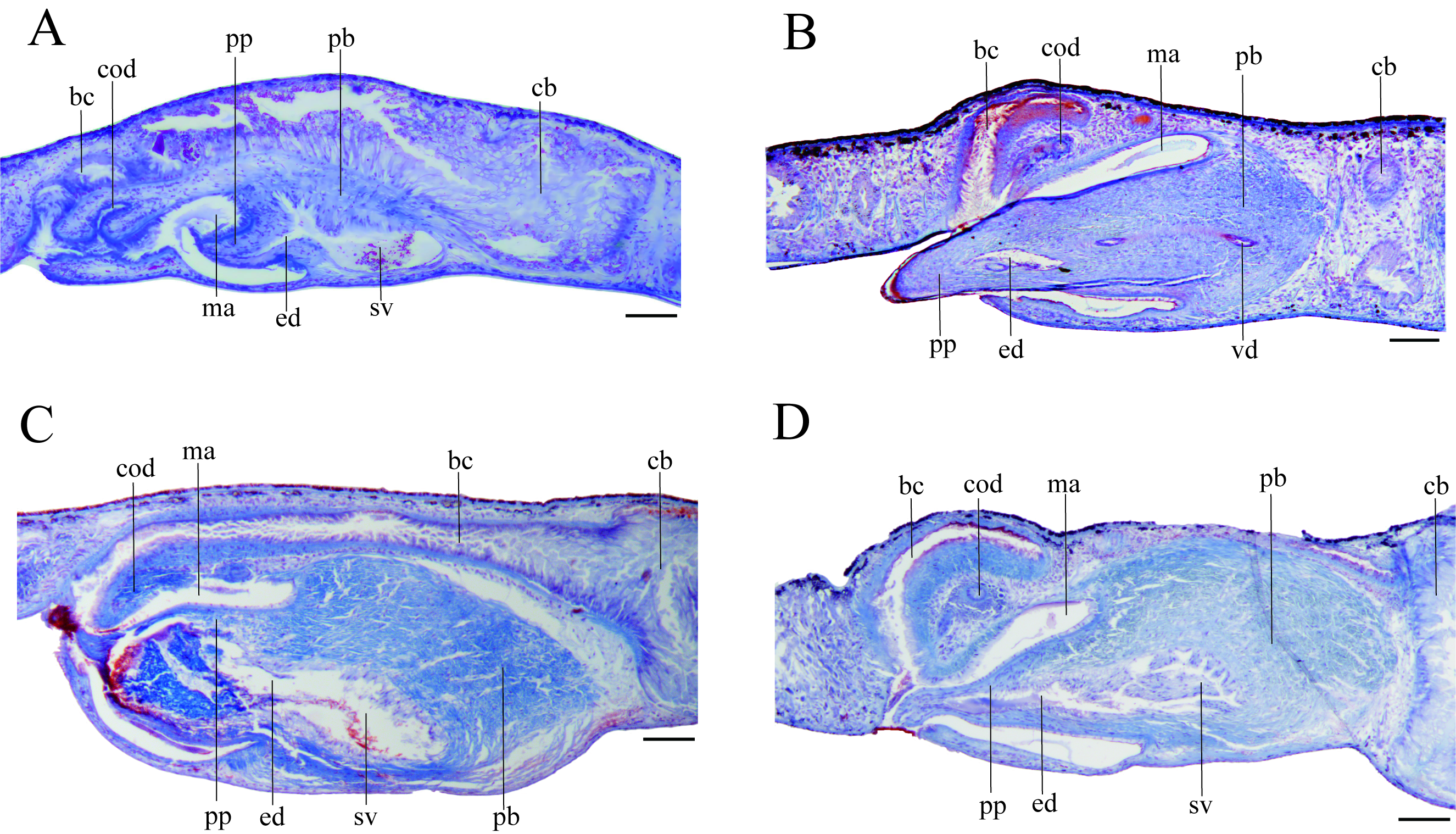

Supplement: Supplementary file 3 — Supplementary Material 3. [file 12864_2025_12467_MOESM3_ESM.tif]

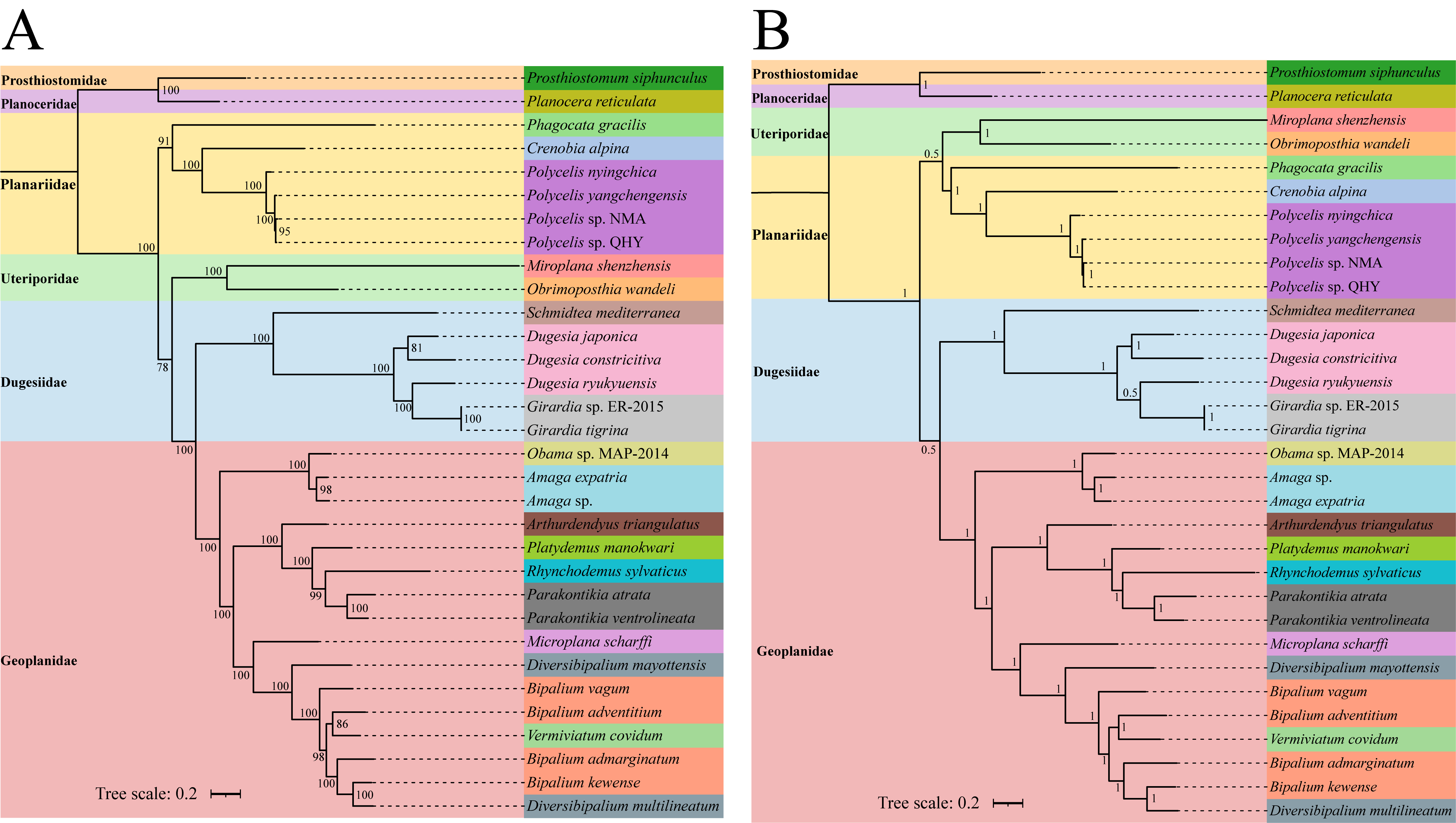

Supplement: Supplementary file 14 — Supplementary Material 14. [file 12864_2025_12467_MOESM14_ESM.tif]
